# A Comparative Study of Treatment With External Fixator Versus Antibiotic Coated Intramedullary Nail in Infected Non-union Long Bones

**DOI:** 10.7759/cureus.29659

**Published:** 2022-09-27

**Authors:** Amandeep S Bakshi, Amandeep Singh, Harsimrat Kaur, Gurleen Kaur, Jaspreet Singh

**Affiliations:** 1 Orthopaedics, Government Medical College & Hospital, Patiala, IND; 2 Medicine and Surgery, Government Medical College & Hospital, Amritsar, IND; 3 Pharmacology, Adesh Medical College & Hospital, Patiala, IND

**Keywords:** antibiotic coated intra-medullary nail, infection, non-union, external fixator, long bones

## Abstract

Introduction: Infected non-union of bones shows problems in providing stability and controlling the infection. The treatment comprises debridement with or without the use of antibiotic cement and achieving stability by either external or internal fixation with or without bone grafting. The use of antibiotic-impregnated cement-coated intramedullary nailing gives stability and treats the infection, thereby permitting early mobilization. External fixation is a definitive mode of fracture stabilization indicated in cases of infected non-unions of the humerus, tibia, and juxta-articular bone. The present study was conducted to compare the results of management of infected non-union long bones treated with an external fixator (Ilizarov and Limb Reconstruction System (LRS)) and vancomycin-coated intramedullary nail.

Materials and method: The present prospective study was conducted on 40 study subjects aged >18 years, and were randomly divided using the alternative method into two groups. Group A: 20 Patients were treated with antibiotic-coated intramedullary interlocking nail (ACIIN). Group B: 20 patients were treated with the external fixator (LRS/Ilizarov). All patients were then evaluated post-operatively using laboratory investigations, and for any sign of inflammation or infection. Results were evaluated using association for the study and application of methods of Ilizarov (ASAMI) scoring system. Data were analyzed using the Statistical Package for Social Sciences (SPSS) version 20 (IBM Corp., Armonk, NY, USA).

Results: It was observed that 80.0% of patients in Group A, and 70% of subjects in Group B showed bony union, revealing a statistically significant (p-value<0.05) difference. Infection control, limb length discrepancy, and deformity were also assessed in both groups. Bone and functional outcomes were assessed using the ASAMI score in both groups. In LRS/Ilizarov, it was observed that 55% of subjects had no infection, 15% showed loosening, and 30% had an infection.

Conclusion: The ACIIN should not be used for bone defects more than 4 cm as it does not achieve stability and for these cases, procedures like external fixators (Ilizarov and LRS) should be used. Antibiotic-coated nail eliminated the complications of the external fixators like pin loosening, and pin infections and was technically less demanding. In our study, stiffness of adjacent joints was more in external fixators (LRS/Ilizarov) than ACIIN.

## Introduction

Infected non-union of bones is a state of failure of union and persistent infection at the fracture site for six to eight months. It is a big challenge presenting huge trouble for the surgeons in terms of cost and time elapsed in the treatment [1]. The prevalence rate is reported to be around 200 long bone non-union cases per annum per million population [2]. The etiological factors are loss of soft tissue, open fracture, pathological fracture, infection of bone after internal fixation, surgical debridement, and chronic osteomyelitis [3]. Infected non-union of bones shows problems in providing stability and controlling the infection. Multiple attempts of failed surgeries lead to fibrosis and shortening of bone, exhaust bone graft donor sites, and also weaken the morale of the patient [4].

The treatment comprises debridement with or without the use of antibiotic cement, prescribing systemic antibiotics, and achieving stability by either external or internal fixation with or without bone grafting [5]. Various studies have advocated the use of antibiotic-impregnated cement-coated intramedullary nailing [6-8]. The non-union rate after using the intramedullary nailing varies according to anatomical location; 0% to 4% in case of tibia fracture [9], 0% to 14% in femoral diaphyseal fractures [10], and 0% to 50% in the humerus [11]. It has been advocated that the cement nail gives stability around the fracture site, and antibiotic cement also permits a higher antibiotic concentration at the local site for around 36 weeks as compared to systemic antibiotics and also has lesser side effects [12]. Thus, this method gives stability and treats the infection, thereby permitting early mobilization, has better performance, and is cost-effective. Antibiotics used should have properties like heat stability, a broad spectrum of activity, and low allergenicity. Gentamicin and vancomycin are the most widely used antibiotics [13].

External fixation is a definitive mode of fracture stabilization indicated in cases of infected non-unions of the humerus, tibia, and juxta-articular bone [14]. It involves the placement of pins or screws into the bone on both the sides of fracture from the outside of the skin using a series of clamps and rods called an external frame [10]. The Ilizarov apparatus is one of its types, used to lengthen or reshape the limb bones through adjustable nuts [15].

With this background, the present study was conducted to compare the results of management of infected non-union long bones treated with an external fixator (Ilizarov and LRS) and vancomycin-coated intramedullary nail.

## Materials and methods

The present randomized prospective study was conducted in the Department of Orthopaedics, Government Medical College, Patiala, Punjab, India on 40 study subjects aged >18 years, having infected non-union long bones with a fracture at diaphysis, but with normal neurological and vascular status. Patients who were pregnant, or had auto-immune disorders, neoplasia, mental health issues, were on immunosuppressive drug therapy, skeletally immature, and unwilling to participate in prolonged post-operative rehabilitation were excluded from the study. Written informed consent was taken from all patients and the study was conducted after taking approval from the Institutional Ethics Committee (IEC No. Trg.9(310)2020/10854).

All subjects were evaluated pre-operatively and were then randomly divided using the alternative method into two groups. Group A: 20 patients were treated with antibiotic-coated intramedullary interlocking nail (ACIIN). Group B: 20 patients were treated with the external fixator (LRS/Ilizarov).

Vancomycin and vancomycin mixed with gentamycin coated intramedullary interlocking nail was used as it has broad spectrum activity, heat stability, and good elution properties. In the external fixator group, the patients underwent Ilizarov or LRS fixators according to the demand of surgery. Thorough debridement of the infected bone and soft tissue was done using copious lavage. After that intramedullary canal was adequately cleaned and prepared to fit the larger diameter nail using standard procedure.

An appropriate size of the antibiotic-impregnated interlocking nail was prepared on a separate sterile table. The intramedullary interlocking nail was coated with bone cement up to 1 mm less than the last reamer. A plastic tube of internal diameter the same as the desired diameter of the cement-coated nail was then filled with a doughy mixture of antibiotics and cement. The nail was then pushed through this tube and allowed to set for 10 to 15 minutes. The plastic tube was then cut open using a surgical knife to retrieve the intramedullary interlocking nail uniformly coated with antibiotic cement. Holes in the locking wall were kept uncovered.

The nail was then inserted in an antegrade fashion. Nail cement debonding during insertion was avoided by allowing inadequate time for the cement to set and bond with the nail and by making small serrations on the surface of the nail.

All patients were evaluated post-operatively for any sign of inflammation or infection using laboratory investigations. Systemic antibiotics were prescribed and physiotherapy was started depending upon the status of fracture healing. All the patients were followed up for a period of one year or till bony union, whichever was earlier.

The results were evaluated using the ASAMI scoring system from their last visit. The ASAMI scoring system was described by Paley et al. as where results varied from excellent (bony union without infection and stiffness of joints) to poor (non-union with infection with deformity) [16].

All the data were collected on a pre-designed proforma. Data were analyzed by appropriate statistical tests using the Statistical Package for Social Sciences (SPSS) version 20 (IBM Corp., Armonk, NY, USA). A p-value of <0.05 was considered significant. Descriptive analysis was done and the mean, standard deviation, and frequencies were calculated.

## Results

The present study revealed that the maximum number of patients (50%) in Group A were 41 to 60 years of age, and 21 to 40 years of age in Group B (50%), with male predominance in both groups. It was observed that maximum patients (70% and 80%) had tibia involvement in Group A and B, respectively, with right side predominance. The most common mode of injury was road traffic accidents observed in 80% and 65% of patients in both Group A and B, respectively.

It was observed that 70% of patients in Group A had undergone surgery once, and 80% from Group B had undergone surgical intervention twice. The mean duration of infection, bony union, bony defect, and shortening were evaluated in both groups (Table 1).

**Table 1:** Mean of various parameters in both the groups *p-value <0.05 is significant ACIIN: Antibiotic-coated intramedullary interlocking nail, LRS: Limb reconstruction system

Parameters	Group A (ACIIN)	Group B (LRS/Illizarov)	Statistical Analysis	
	Mean ± SD	Mean ± SD	t-test	p-value
Duration of infection (months)	11.05 ± 6.78	10.2 ± 7.8	2.223	0.052*
Duration of bony union (weeks)	26.252 ± 5.731	31.75 ± 4.012	2.781	0.021*
Bone defect (cm)	2.15 ± 2.02	5.7 ± 2.87	3.005	0.007*
Shortening (cm)	1.576 ± 0.43	1.85 ± 0.25	0.997	0.188*

The type of previous surgery and present treatment was noted in both groups (Tables 2-3).

**Table 2:** Distribution of study subjects according to the type of previous surgery done in both groups ORIF: Open reduction with internal fixation, ACIIN: Antibiotic-coated intramedullary interlocking nail, LRS: Limb reconstruction system

Previous surgery done	Group A (ACIIN)	Group B (LRS/Ilizarov)
	Frequency (%)	Frequency (%)
Closed reduction with Intra-medullary nail	2 (10)	1 (5)
Closed reduction with External fixator	2 (10)	1 (5)
Debridement with Plating	-	1 (5)
Debridement with Intra-medullary nail	-	4 (20)
Debridement with External fixator	5 (25)	2 (10)
ORIF with Intra-medullary nail	6 (30)	6 (30)
ORIF with Plating	5 (25)	5 (25)
Total	20 (100)	20 (100)

**Table 3:** Distribution of study subjects according to treatment done in both the groups ACIIN: Antibiotic-coated intramedullary interlocking nail, LRS: Limb reconstruction system

Treatment	Bony defect (cm)	Number of cases treated	Number of cases showing bony union	Percentage
Debridement with ACIIN	1-2	13	13	85
Debridement with ACIIN and bone grafting	3-4	7	4	
Debridement with LRS/Ilizarov	5-8	20	16	80

Ankle and knee mobility was also assessed (Table 4).

**Table 4:** Distribution of study subjects according to ankle and knee mobility in both groups *p-value <0.05 is significant ACIIN: Antibiotic-coated intramedullary interlocking nail, LRS: Limb reconstruction system

	Knee mobility		Ankle mobility	
	Group A (ACIIN)	Group B (LRS/Ilizarov)	Group A (ACIIN)	Group B (LRS/Ilizarov)
	Frequency (%)	Frequency (%)	Frequency (%)	Frequency (%)
Normal	18 (90)	15 (75)	18 (90)	15 (75)
Stiff	2 (10)	5 (25)	2 (10)	5 (25)
Total	20 (100)	20 (100)	20 (100)	20 (100)
Chi-square test	10.670		7.589	
p-value	0.049*		0.005*	

It was observed that 80% of patients in Group A and 70% of subjects in Group B showed a bony union, revealing a statistically significant (p-value <0.05) difference. Infection control, limb length discrepancy, and deformity were also assessed in both groups (Table 5).

**Table 5:** Distribution of study subjects according to post-operative assessment parameters in both the groups *p-value <0.05 is significant ACIIN: Antibiotic-coated intramedullary interlocking nail, LRS: Limb reconstruction system

Infection control	Group A (ACIIN)	Group B (LRS/Ilizarov)
	Frequency (%)	Frequency (%)
Controlled	16 (80)	14 (70)
Not controlled	4 (20)	6 (30)
Total	20 (100)	20 (100)
Chi-square test	8.835	
p-value	0.025*	
Limb length discrepancy (LLD)	Group A (ACIIN)	Group B (LRS/Ilizarov)
	Frequency (%)	Frequency (%)
Same as before	12 (60)	2 (10)
No	-	8 (40)
Yes	8 (40)	10 (50)
Total	20 (100)	20 (100)
Chi-square test	5.041	
p-value	0.021*	
Deformity	Group A (ACIIN)	Group B (LRS/Ilizarov)
	Frequency (%)	Frequency (%)
<7 degree	16 (80)	14 (70)
>7 degree	4 (20)	6 (30)
Total	20 (100)	20 (100)
Chi-square test	3.332	
p-value	0.08*	

Bone and functional outcomes were assessed using the ASAMI score in both groups (Table 6).

**Table 6:** Distribution of study subjects according to the ASAMI score on bone and functional outcomes in both groups *p-value <0.05 is significant ACIIN: Antibiotic-coated intramedullary interlocking nail, LRS: Limb reconstruction system, ASAMI: Association for the study and application of methods of Ilizarov

	ASAMI Score (Bone outcome)		ASAMI Score (Functional outcome)	
	Group A (ACIIN)	Group B (LRS/Ilizarov)	Group A (ACIIN)	Group B (LRS/Ilizarov)
	Frequency (%)	Frequency (%)	Frequency (%)	Frequency (%)
Excellent	13 (65)	12 (60)	13 (65)	12 (60)
Good	1 (5)	2 (10)	3 (15)	6 (30)
Fair	2 (10)	3 (15)	1 (5)	0
Poor	4 (20)	3 (15)	3 (15)	2 (10)
Total	20 (100)	20 (100)	20 (100)	20 (100)
Chi-square test	1.005		1.108	
p-value	0.079*		1.914*	

In LRS/Ilizarov, it was observed that 55% of subjects had no infection, 15% showed loosening, and 30% had an infection. In Group B, among four patients with a habit of smoking, each had bony union and persistent infection. Among patients with diabetes mellitus, four had bony union and three had a persistent infection. In Group A, among four patients with a habit of smoking, each had a bony union and persistent infection. Among patients with diabetes mellitus, two had bony union and one had a persistent infection. The distribution of infective organisms was evaluated in both groups (Table 7).

**Table 7:** Distribution of infective organisms in both groups ACIIN: Antibiotic-coated intramedullary interlocking nail, LRS: Limb reconstruction system

Infective organism	Group A (ACIIN)	Group B (LRS/Ilizarov)
Staphylococcus aureus	15	13
Klebsiella	0	1
Pseudomonas aeruginosa	2	3
Mixed flora	2	2
Escherichia coli	1	1

## Discussion

The treatment of infected non-union of long bones requires radical debridement of the sinus tracts, dead bone, and fibrous tissue till the paprika sign appears and rigid fixation is achieved. The use of local antibiotics is more beneficial than systemic, in terms of reducing the side effects [17].

In the present study, we observed that infection of long bones was mainly caused by *Staphylococcus aureus*, which was in accordance with the studies done by Bhatia et al. [18] and Thonse et al. [6]. To eliminate such infections, broad-spectrum antibiotics should be used. In the present study, we used vancomycin and a combination of vancomycin and gentamicin as antibiotics because it has heat-stable physical properties and is an ideal antibiotic for loading bone cement. Vancomycin is bactericidal and acts by inhibiting the synthesis of the cell wall. Similar to our study, many studies used the combination of vancomycin and gentamicin or teicoplanin [8,19].

Similar to our study, Shyam et al. [20] conducted a study using the ACIIN for the treatment of infected non-union long bones. They concluded that ACIINs are useful for infection control and bone union in cases of infected non-union with bone defect <6 cm. They advocated that in cases with defects>6 cm, other alternatives should be used. Similarly, Bhatia et al. [18] also studied the role of antibiotic cement-coated nailing in infected non-union of the tibia. Bony union was achieved in 60% of patients having bone defects less than 2 cm with an average time of union at 32 weeks. The remaining patients with bone defects of more than 2 cm required procedures such as bone grafting or exchange nailing. They observed different complications like difficulty in nail removal, broken nail, bent nail, and recurrence of infection. Maini et al. [21] used Ilizarov in 4 cm to 15 cm bone defects and achieved effective bone union. Infection control with antibiotic-coated intramedullary interlocking nail (ACIIN) was 80%. Thonse et al. [6] also achieved infection control in 84% of cases.

Various studies reported that external fixators are associated with complications such as a high incidence of pin infections, pin loosening, poor compliance of patients, joint stiffness, and muscle contractures [22,23]. In accordance with these studies, our present study reported pin loosening in 15%, pin infection in 30%, and joint stiffness in 25% of patients. The average duration of bony union was noted as 31 weeks.

The ASAMI scoring was used to evaluate the bone and functional outcomes in the studies mentioned above (Table 8). The results of our study were comparable with the above studies (Table 9) [24-27].

**Table 8:** Comparison between the results of different studies using the ASAMI (bone scoring) system ACIIN: Antibiotic-coated intramedullary interlocking nail, LRS: Limb reconstruction system, ASAMI: Association for the study and application of methods of Ilizarov

Authors	Patients (n)	Frame	Results			
			Excellent (%)	Good (%)	Fair (%)	Poor (%)
Rohilla et al.[23]	35	Ilizarov	60	34.3	0	5.7
Maini et al.[20]	40	Ilizarov	70	10	0	20
Khan A et al.[24]	20	LRS	75	10	5	10
Gaurav K et al. [25]	30	LRS	73.33	13.33	6.67	6.67
Present study	20	LRS/ Ilizarov	60	10	15	15
	20	ACIIN	65	5	10	20
Balasubramaniam et al. [26]	20	ACIIN	60	10	5	25

**Table 9:** Comparison between the results of different studies using the ASAMI (functional scoring) system ACIIN: Antibiotic-coated intramedullary interlocking nail, LRS: Limb reconstruction system, ASAMI: Association for the study and application of methods of Ilizarov

Authors	Patients (n)	Frame	Results			
			Excellent (%)	Good (%)	Fair (%)	Poor (%)
Rohilla et al. [23]	35	Ilizarov	45.7	48.5	2.9	0
Maini et al. [20]	40	Ilizarov	27	40	10	23
Khan A et al. [24]	20	LRS	60	20	10	10
Gaurav K et al. [25]	30	LRS	53.33	23.33	16.67	6.67
Present study	20	LRS/ Ilizarov	60	30	0	10
	20	ACIIN	65	15	5	15
Balasubramaniam et al. [26]	20	ACIIN	63.6	18.2	-	18.2

Hernigou et al. [28] did a study that mentioned that smoking is a predictor of a negative outcome in diaphyseal fracture healing and has a significant effect on the non-union of bones. Gortler et al. [29] concluded that diabetes substantially alters bone metabolism and soft tissue healing, posing a risk of adverse fracture healing and other complications. They revealed that the presence of diabetes can significantly increase the risks of infection, malunion, non-union, and re-operation across a wide variety of surgically treated lower extremity fractures. This study provides prognostic information for clinicians and may aid in guiding treatment for the study population.

The limitation of our study is the small sample size, and so we could not completely remove all the confounding variables. The relative rarity of cases with infected non-unions did not allow us to have a greater number of cases. These cases formed a heterogeneous group. Also, a variable amount of antibiotic in bone cement was used in the ACIIN group which depended on the canal diameter of the bone in which the nail was being inserted.

## Conclusions

The antibiotic nail is a single-stage procedure achieving both union and infection control for bone defects less than 4 cm. As per our study, ACIIN should not be used for bone defects more than 4 cm as it does not achieve stability and for these cases, procedures such as external fixators (LRS/Ilizarov) should be used. Antibiotic-coated nail eliminated complications of the external fixators like pin loosening and pin infections and was technically less demanding. In our study, stiffness of adjacent joints was more in external fixators (LRS/Ilizarov) than in ACIIN. Infection control was marginally better in ACIIN than in external fixators. As per our study bony union was seen earlier with ACIIN than external fixators (LRS/Ilizarov). Smoking and diabetes seem to have a significant effect on delayed and non-union of long bones.
